# Amyotrophic lateral sclerosis diagnosis using machine learning and multi-omic data integration

**DOI:** 10.1016/j.heliyon.2024.e38583

**Published:** 2024-10-01

**Authors:** Hima Nikafshan Rad, Zheng Su, Anne Trinh, M.A. Hakim Newton, Jannah Shamsani, NYGC ALS Consortium, Abdul Karim, Abdul Sattar

**Affiliations:** a School of Information and Communication Technology, Griffith University, 170 Kessels Rd, Nathan, Brisbane, 4111, QLD, Australia; b GenieUs Genomics Pty Ltd, Sydney, 2000, NSW, Australia; c School of Biotechnology and Biomolecular Sciences, Faculty of Science, The University of New South Wales, Sydney, 2052, NSW, Australia; d School of Information and Physical Sciences, The University of Newcastle, University Drive, Callaghan, Newcastle, 2308, NSW, Australia; e The New York Genome Center, 101 Avenue of the Americas, New York, 10013, NY, USA; f Institute of Integrated and Intelligent Systems, Griffith University, 170 Kessels Rd, Nathan, Brisbane, 4111, QLD, Australia

**Keywords:** ALS diagnosis, Pathway level analysis, Variational autoencoder, Multi-omic integration

## Abstract

Amyotrophic Lateral Sclerosis (ALS) is a complex and rare neurodegenerative disorder characterized by significant genetic, molecular, and clinical heterogeneity. Despite numerous endeavors to discover the genetic factors underlying ALS, a significant number of these factors remain unknown. This knowledge gap highlights the necessity for personalized medicine approaches that can provide more comprehensive information for the purposes of diagnosis, prognosis, and treatment of ALS. This work utilizes an innovative approach by employing a machine learning-facilitated, multi-omic model to develop a more comprehensive knowledge of ALS. Through unsupervised clustering on gene expression profiles, 9,847 genes associated with ALS pathways are isolated and integrated with 7,699 genes containing rare, presumed pathogenic genomic variants, leading to a comprehensive amalgamation of 17,546 genes. Subsequently, a Variational Autoencoder is applied to distil complex biomedical information from these genes, culminating in the creation of the proposed Multi-Omics for ALS (MOALS) model, which has been designed to expose intricate genotype-phenotype interconnections within the dataset. Our meticulous investigation elucidates several pivotal ALS signaling pathways and demonstrates that MOALS is a superior model, outclassing other machine learning models based on single omic approaches such as SNV and RNA expression, enhancing accuracy by 1.7 percent and 6.2 percent, respectively. The findings of this study suggest that analyzing the relationships within biological systems can provide heuristic insights into the biological mechanisms that help to make highly accurate ALS diagnosis tools and achieve more interpretable results.

## Highlights

• An advanced machine learning-based multi-omic model was developed to enhance the understanding of ALS mechanisms.
• MOALS combined 9,847 ALS-related genes with 7,699 rare variant genes, creating a comprehensive dataset for precise analysis.
• Findings showed that unsupervised clustering exposed critical ALS pathways, validating MOALS performance in gene analysis.
• The proposed model outperformed existing ML methods, with accuracy improvements ranging from 1.7% to 6.2% in ALS prediction.

## Graphical abstract

**Figure:**
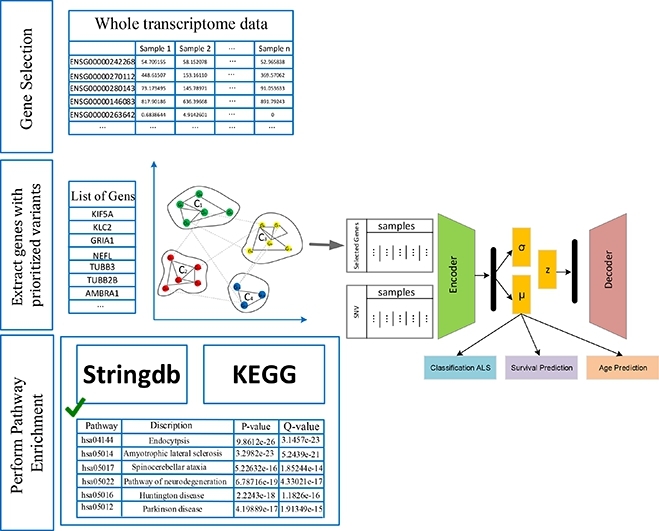

## Nomenclature

**ALS**: Amyotrophic Lateral Sclerosis
**MND**: Motor Neuron Disease
**FDA**: Food and Drug Administration
**GWAS**: Genome-Wide Association Studies
**ML**: Machine Learning
**FALS**: Familial Amyotrophic Lateral Sclerosis
**SALS**: Sporadic Amyotrophic Lateral Sclerosis
**RNA-seq**: RNA Sequencing
**mRNA**: Messenger RNA
**WGS**: Whole Genome Sequencing
**SNV**: Single Nucleotide Variant
**SHAP**: Shapley Additive Explanations
**iPCS**: Induced Pluripotent Stem Cells
**ATAC-seq**: Assay for Transposase-Accessible Chromatin using sequencing
**ChIP-seq**: Chromatin Immunoprecipitation Sequencing
**Hi-C**: A method to study the three-dimensional architecture of genomes
**MOALS**: Multi-Omics for ALS
**VAE**: Variational Autoencoder
**GRCh38**: Genome Reference Consortium Human Build 38
**bwa-mem2**: Burrows-Wheeler Aligner-Maximum Exact Matches 2
**GATK**: Genome Analysis Toolkit
**VEP**: Variant Effect Predictor
**FASTQC**: Fast Quality Control
**FDR**: False Discovery Rate
**BQSR**: Base Quality Score Recalibration
CE: Cross Entropy
**MSE**: Mean Squared Error
**DEM**: Deep Embedding Module
**BCE**: Binary Cross Entropy
**SVM**: Support Vector Machine
**UMAP**: Uniform Manifold Approximation and Projection
**PCA**: Principal Component Analysis
**MTLR**: Multi-Task Logistic Regression
**GradNorm**: Gradient Normalization
**KEGG**: Kyoto Encyclopedia of Genes and Genomes
**ER**: Endoplasmic Reticulum
**UPR**: Unfolded Protein Response
**UPS**: Ubiquitin-Proteasome System
**STRING-db**: Search Tool for the Retrieval of Interacting Genes/Proteins database
**TF-IDF**: Term Frequency-Inverse Document Frequency
**ROC**: Receiver Operating Characteristic
**AUC**: Area Under the Curve
**FCNN**: Fully Connected Neural Network
**RFR**: Random Forest Regression
**SVR**: Support Vector Regression
**RMSE**: Root Mean Square Error
**MAE**: Mean Absolute Error
**MedAE**: Median Absolute Error
R2: Coefficient of Determination
**C-index**: Concordance Index
**IBS**: Integrated Brier Score
**CoxPH**: Cox Proportional Hazards
**AI**: Artificial Intelligence
**RNA**: Ribonucleic Acid

## 1 Introduction

Amyotrophic Lateral Sclerosis (ALS) is a neurodegenerative disease that causes Motor Neuron (MN) loss in the spinal cord and the motor cortex. ALS, also known as Lou Gehrig's disease, leads to progressive paralysis, muscular atrophy, and death. According to the US Centers for Disease Control and Prevention, 12,000 to 15,000 Americans are thought to have ALS [1]. About 10 percent ALS cases are *familial* while the rest 90 percent are *sporadic*. Some monogenic drivers of familial ALS include mutations in the genes *C9orf72*, *SOD1*, *TARDBP*, and *FUS* [2]. However, the pathogenesis of sporadic ALS still has no known genetic or environmental cause. Familial and sporadic ALS patients have few treatment options. Despite 30 years of clinical trials, only Rilutek (riluzole), Radicava (edaravone), Relyvrio (sodium phenylbutyrate and taurursodiol), and Qalsody (tofersen) have been approved by the FDA as symptomatic treatments for ALS (in 1995, 2017, 2022, and 2023 respectively). Unfortunately, drug neither stops the disease nor restores motor function [3].

In understanding the genetic complexities of Amyotrophic Lateral Sclerosis (ALS), it is crucial to consider that known causative genetic variants manifest predominantly in later life and account for only 10 percent of ALS heritability, leaving a large “missing heritability” component that may be polygenic or even omnigenic in nature [4]. Addressing these issues requires a systems biology perspective that considers disease as a dysfunction in biological modules or pathways. Our research addresses these gaps by focusing on the biological pathways disrupted in ALS, employing a two-step approach for mapping genotypes to disease prevalence. In the first stage, genes were clustered based on their expression profiles in specific brain and spinal cord regions of ALS patients, compared to healthy controls. In the second stage, germline mutations and gene expression data were integrated into a general classifier using a multi-omics approach. Our methodology introduces a novel diagnostic process for ALS, addressing critical methodological challenges such as the limitations of genome-wide association studies (GWAS) in capturing non-additive genetic effects like epistasis. These findings open up avenues for further research, particularly in the exploration of machine learning models to capture the complexity of gene-gene interactions and the potentially omnigenic nature of ALS.

Machine learning (ML) as a data-driven platform has played a significant role in making progress in the diagnosis of many diseases, including ALS. However, particularly for ALS, mainly single-omic approaches have been used in ML models for diagnoses [4], [7], [9], prognosis [12], mutation [11], subtyping [6], biomarkers [10], pathway [5], biological identification [13], and gene discovery [8]. Another ML approach RefMap [14] uses iPCS cells, ATAC-seq, Histone ChIP-seq, Hi-C, and RNA-seq for gene discovery. Further details about the mentioned studies and their specific features have been summarized in Table 1 (top).

**Table 1:** Literature review of ML based methods for ALS (top) and other diseases (bottom).

Ref	Disease	Omics	Approach	ML model	Feature
[5]	ALS	mRNA expression	Pathways	Unsupervised hierarchical clustering	Classification SALS and control, Identification of common pathogenic link between FALS and SALS
[6]	ALS	RNA-seq	Subtyping	Clustering	Identification of TARDBP/TDP-43 and retrotransposon expression two factors for ALS
[7]	ALS	Whole Genome Sequence	Diagnosis	Convolutional neural network, Deep neural network	Identification of ALS-associated promoter regions, ALS classification
[8]	ALS	Protein–protein interaction data, gene function annotation, known disease-gene associations	Gene discovery	knowledge-based machine learning	Gene prioritization for ALS
[9]	ALS	RNA expression	Diagnosis	Deep convolution neural networks and Shapley Values	ALS classification
[10]	ALS	Plasma samples	Biomarker	Random Forest	Presenting very high prediction rates for ALS diagnosis and prognosis
[11]	ALS	Whole Genome Sequence	Mutation	Unsupervised machine-learning	Identification of subset of common genetic variants for ALS
[12]	ALS	Demographic, Family history, Genetic factor	Prognosis	Probabilistic Causal Discovery	Assess genetic factors association ALS clinical progressions
[13]	ALS	Multichannel fluorescence microscopy data	Biological Identification	Image-based deep learning	Evaluation the impact of stress on valosin-containing protein related to ALS
[14]	ALS	iPCS cells, ATAC-seq, Histone ChIP-seq, Hi-C, RNA-seq	Diagnosis	Regional fine-mapping (RefMap)	Identification of risk genes related to ALS
[4]	ALS	Whole Genome Sequence	Diagnosis	capsule networks	Disease prediction from individual genotype profiles

[15]	Cancer	mRNA expression, DNA methylation, microRNA expression	Diagnosis	Variational Autoencoder	Implementation of multi-task platform for cancer diagnosis
[16]	Cancer, Alzheimer	mRNA, DNA methylation, RNA expression	Biomarker	Graph convolutional networks	Identification of important biomarkers
[17]	Cancer	mutations, copy number changes, DNA methylation, gene expression	Biomarker	Graph convolutional networks	Identification of new cancer genes and their associated molecular mechanisms
[18]	Cancer	mRNA expression, DNA methylation, microRNA expression	Diagnosis	Interpretable deep learning, Variational Autoencoder	Discovering of biomedical knowledge, cancer classification
[19]	Cancer	DNA methylation, gene expression	Translation framework	Generative adversarial networks	Omics-to-omics translation

So far single-omic based approaches have led to significant progress in ML-based diagnosis of ALS. For instance, [4] made a groundbreaking advancement in ALS research by being the first to utilize capsule networks on a whole-genome scale, achieving an unprecedented 86.9 percent predictive accuracy and illuminating ‘non-additive’ genes that have previously remained obscured in linear models. On the other hand, in an investigation developed by [9], the convolutional neural networks and the Shapley Additive Explanations (SHAP) as a novel paradigm shift by converting RNA expression values into pixel-based images for analysis, yielding 80.7 percent accuracy and a level of interpretability that allowed for the identification of disease-critical genes. Moreover, a two-step deep convolutional neural network approach utilized by [7] underscores the importance of domain-specific architecture, offering a 77 percent accuracy rate in ALS prediction and highlighting the value of incorporating prior genomic knowledge into machine learning models. It should be mentioned that single-omic techniques have their deficiency in terms of integrating various methodologies, and they face challenges in exploring genetic, biochemical, metabolic, proteomic, and epigenetic mechanisms that are important underlying factors for ALS. To reach a comprehensive presentation of various layers of regulation, interconnected complexity and higher resolution picture in biological systems, the multi-omic approach might be useful. To this end, incorporation of multi-omic approaches within ML models provides a powerful analytical option that is capable of finding patterns in dense datasets for genomically heterogeneous and complex diseases [15], [18], biomarker identification [16], [17], and other applications [19].

In an interdisciplinary effort to address the prevailing challenges of omics data analysis in the biomedical sector, we introduce a compendium of state-of-the-art computational methods. For example, [18] utilized the framework of XOmiVAE to address the pressing need for explainability in deep learning applications, particularly in cancer classification, by elucidating the contributions of individual genes and latent dimensions. Complementing this, [17], introduced an integrated EMOGI multi-omics pan-cancer data with protein–protein interaction networks through graph convolutional networks, thereby providing an accurate and interpretable model for predicting cancer genes. In another study, [16], presented MOGONET offers advancements in the realm of multi-omics integrative analysis, excelling in both data classification and biomarker discovery across disparate biomedical applications. To confront the challenges associated with high-dimensional data and cross-omics translation [15] and [19] implemented OmiEmbed and OmiTrans. This action extended the boundaries of current computational capabilities. However, all the mentioned efforts have been restricted to some specific ones to cancers, and Alzheimer and ALS diagnosis by multi-omics approach still has not been taken into account. Further details about the multi-omics studies and their specific features in other diseases have been reported in Table 1 (bottom).

In this paper, for the first time, a multi-omics approach based on ML for ALS diagnosis, first symptom and survival prediction is presented. We use unsupervised clustering to provide an interpretable biological processes on gene expression profiles to identify 9847 genes associated with ALS pathways, which were then integrated with 7,699 genes that include rare, predicted pathogenic genomic variants prioritized based on biological knowledge. We use a variational autoencoder to capture biomedical information in the integrated 17,546 genes. We named our model Multi-Omics for ALS (MOALS). Our investigation detects several ALS signaling pathways, and MOALS outperforms other existing ALS classification models in accuracy.

In summary, the contributions of this paper are as follows:

• Acquiring and preprocessing whole genome sequencing (WGS) data to extract and analyze single nucleotide variants (SNVs) based on biological knowledge.
• Selecting features using a clustering algorithm for ALS pathway-level analyses on mRNA transcriptomes.
• Combining the above two in a variational autoencoder learning framework to predict ALS status, the age of first symptoms and survival predictions.

## 2 Materials and methods

**Figure 1:**
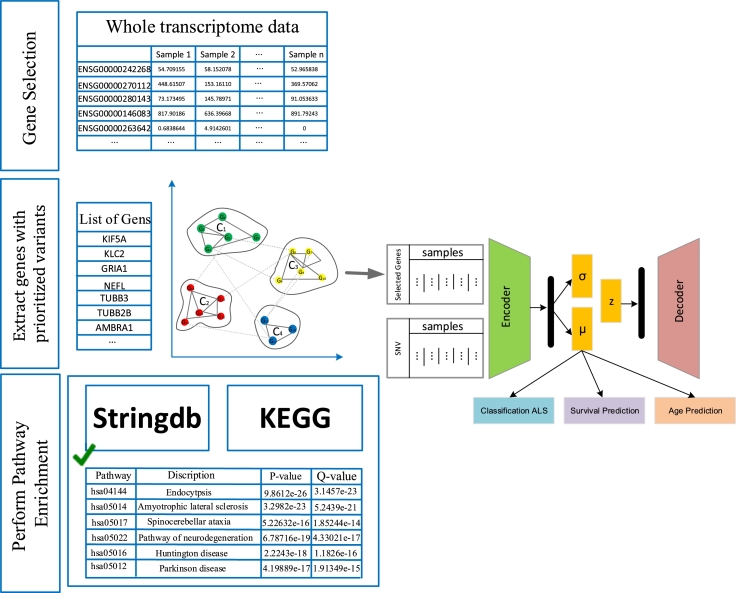
The overview of the implemented method for ALS diagnosis.

In this section, the followed methodology for feature selection, the ML platform, and the pathways level analysis will be described. The innovative platform named MOALS is designed for multi-omic data processing activities. MOALS's workflow may be broken down into three components: (1) Pre-processing and extraction of genes in ALS pathways by clustering of gene expression profiles; To begin, transcriptome data were pre-processed, and feature preselection was performed to identify ALS pathway signaling and to eliminate noise in the expression data which may degrade classification task performance. (2) Then, in the variations extraction step, the single nucleotide variants (SNV) were detected by the sequence alignment/map tools, and the variants were prioritized based on the biological knowledge using bioinformatic programs. Briefly, fastq raw data was aligned to human reference genome (GRCh38) using bwa-mem2. SNVs, and small insertions and deletions (indels) were then called using GATK (version 4.2.5.0). Next, SNVs and small indels were annotated with gene information using VEP (Version 104.2). Finally, variants were filtered and ranked using custom scripts. (3) Machine learning using Multi-omics data; The initial class probability estimates from each single omic were used to compute multi-omics integration using variational autoencoder based on concatenating each omics layer. In the following sub-sections, detailed explanations related to each part will be presented. The overview scheme relating to each step has been depicted in Fig. 1.

### 2.1 Data acquisition and preprocessing

The sequencing (RNA and WGS) analyzed in this study from the Target ALS cohort were obtained upon application to the New York Genome Center with the data request. The selection of 672 cases, including 593 ALS and 79 non-ALS cases, was based on the availability of high-quality multi-omics data necessary for robust analysis. Due to the limited availability of Control samples, we selected a robust model specifically designed to effectively handle the non-balanced nature of the data.

#### 2.1.1 RNA-sequencing analysis

The raw RNA sequencing data was processed in-house according to the following pipeline. We used FASTQC [20] to perform quality control after obtaining the raw sequencing data in fastq format, with mean quality value across each base location in the read and per-sequence quality scores as the major criteria for data quality evaluation. Kallisto [21] pseudo-aligned the sequences to the reference genome of GRCh38 from Ensembl release 95 [22].

To conduct pathway analysis, GO annotations and homology information was obtained from Ensembl BioMart database [23]. Enrichr [24] was used to perform gene set enrichment analysis. For the pathway analysis, we utilized the Kyoto Encyclopedia of Genes and Genomes (KEGG), a comprehensive database resource that offers a systematic understanding of biological functions and the interconnection of various elements of the biological system. KEGG pathway annotations were employed against the whole genome as a background reference to identify statistically significant pathways. A false discovery rate (FDR) cutoff 0.05 was used to select significantly enriched pathways.

#### 2.1.2 Genomic variant extraction

Whole-genome sequencing (WGS) data, represented by raw fastq files, were meticulously aligned to the GRCh38 reference genome, employing the GATK best-practices workflow. This comprehensive workflow incorporated BWA-MEM for alignment, Picard tools for annotating repetitive reads, local realignment surrounding indels, and a base quality score recalibration (BQSR) [25] to refine alignment accuracy.

Following the precise alignment, individual sample variant calling was executed utilizing HaplotypeCaller. This was augmented by joint genotyping and Variant Quality Score Recalibration to enhance the reliability of variant identification. Subsequently, the generated variants underwent rigorous annotation using Variant Effect Predictor (VEP) [26] and bcftools [27] to delineate the variant's potential impacts, focusing on those with predicted effects on protein-coding sequences, as discerned through functional annotation.

Post annotation, a series of meticulous filtering steps were instigated. This involved isolating variants associated with canonical transcripts, recognized gene symbols, and variants demonstrating a population allele frequency < 0.01 or those absent in large normal populations [26]. With the acquired biological context from extensive annotation, the variants were systematically scored and ranked based on their predicted impacts, prioritizing variants such as frameshift, stop gained, transcript ablation, stop lost, start lost, transcript amplification, and splice donor and acceptor variants. These were meticulously ranked with scores of 5, 3, and 2, to delineate their relative significance. After evaluating all samples related to patients, 7699 variants of interest were identified. The number of repetitions for each variant in each individual were different. This exhaustive and systematic approach ensured the meticulous identification and evaluation of variants with profound functional implications, enabling a holistic insight into the investigated genomic landscapes.

### 2.2 Feature selection based on pathway-level analyses

Fuzzy k-means clustering is utilized as a central technique for grouping mRNA gene expression data to discern intricate patterns associated with Amyotrophic Lateral Sclerosis (ALS). Unlike traditional k-means clustering, which assigns each data point rigidly to a single cluster, fuzzy k-means permits a data point to belong to multiple clusters with varying degrees of membership. This fuzziness is crucial in revealing complex patterns in gene expression data, particularly in cases where the boundaries between different expression levels are not distinctly defined.

The primary advantage of using fuzzy k-means for gene expression analysis is its ability to handle the inherent uncertainty and variability in gene expression levels. This provides a nuanced and comprehensive understanding of the underlying biological phenomena, enabling more biologically meaningful interpretations of the heterogeneous nature of mRNA transcripts associated with ALS. The flexibility of fuzzy k-means is particularly relevant in addressing the complex nature of neurodegenerative disorders such as ALS, thereby facilitating more refined and precise analyses compared to conventional clustering methods.

To enhance the description and reproducibility of our clustering process, we detail our methodology as follows:

• Mathematical Formulation: The fuzzy k-means algorithm assigns membership degrees using the formula shown in Equation (1):
  (1) $$u_{ij}=\frac{1}{\sum_{k=1}^{c}{\left(\frac{\left\|x_{i}-v_{j}\right\|}{\left\|x_{i}-v_{k}\right\|}\right)}^{\frac{2}{m-1}}},$$
  where $u_{ij}$ is the membership degree of the *i*-th data point in the *j*-th cluster, $x_{i}$ is the *i*-th data point, $v_{j}$ is the centroid of the *j*-th cluster, *c* is the number of clusters, and *m* is the fuzziness parameter.
• Cluster Initialization: We employed the fuzzy k-means algorithm with a specified range of cluster counts (6 to 12 clusters) to determine the optimal granularity for our data.
• Cluster Optimization: The algorithm iteratively adjusts the cluster centers based on a weighted average of the data points, where weights correspond to the degree of belonging of each point to a particular cluster. The process continues until the changes in the cluster centers are minimal, ensuring convergence.
• Membership Degree Evaluation: Each gene's membership degree to the cluster centers was evaluated to score and rank the genes based on their centrality. This scoring influenced their subsequent selection for pathway analysis.
• Statistical Evaluation: Ranked genes were analyzed through pathway-level enrichment tests to determine their biological significance, enhancing our understanding of ALS-associated pathways.

By providing these additional details, we aim to improve the transparency of our analytical approach and allow for better reproducibility of our results by other researchers in the field.

### 2.3 Network architecture

The MOALS platform integrates a multi-task deep learning framework to analyze multi-omics data, which is crucial for applications like ALS classification, predicting the age at first symptoms, and survival prediction. Central to this framework is the Deep Embedding Module (DEM), which employs a Variational Autoencoder (VAE) to transform high-dimensional multi-omics data into a meaningful, low-dimensional latent space.

#### 2.3.1 Variational Autoencoder (VAE) overview

The VAE is a cornerstone of our DEM, offering a robust method for learning deep representations of complex datasets. Unlike traditional autoencoders, VAEs introduce a probabilistic approach to encode inputs into a latent (hidden) space. This approach not only helps in compressing the data but also in generating new data points, hence facilitating the modeling of complex biological phenomena.

##### Mathematical framework of VAE

In the VAE, each high-dimensional input vector $x^{\left(i\right)}\in R^{d}$ from the multi-omics dataset D is mapped to a latent vector $z^{\left(i\right)}\in R^{p}$, where p≪d. The mapping is done through a probabilistic encoding process defined by a distribution $q_{\phi }(z|x)$, typically assumed to be Gaussian, as shown in Equation (2):

(2) $$q_{\phi }(z|x)=N(z;\mu (x),\sigma {\left(x\right)}^{2}I)$$

where μ(x) and σ(x) are outputs from the encoder network, parameterized by *ϕ*, representing the mean and variance of the latent distribution.

The VAE optimizes the parameters by maximizing the Evidence Lower Bound (ELBO) to the logarithm of the likelihood of the data, as shown in Equation (3):

(3) $$L(\phi ,\theta ;x^{\left(i\right)})=E_{q_{\phi }(z|x^{\left(i\right)})}[\log p_{\theta }(x^{\left(i\right)}|z)]-D_{KL}(q_{\phi }(z|x^{\left(i\right)})||p(z))$$

where $p_{\theta }(x|z)$ is the probability of reconstructing *x* given *z*, modeled by the decoder network with parameters *θ*, and $D_{KL}$ represents the Kullback–Leibler divergence, encouraging the encoded latent variables to approximate a prior distribution p(z), typically a standard normal distribution.

This ELBO component ensures that the VAE not only reconstructs the data efficiently but also regularizes the learning process to avoid overfitting, making the model robust to unseen data.

##### Implementation details

In our implementation, the VAE's encoder and decoder networks consist of fully connected layers, with non-linear activation functions like ReLU to introduce non-linearities into the model, crucial for capturing complex patterns in the data. The encoder compresses the input into the latent variables *μ* and *σ*, and the decoder reconstructs the input from the latent representation sampled using the reparameterization trick, as shown in Equation (4):

(4) z=μ+σ⊙ϵ,ϵ∼N(0,I)

This step ensures that gradients can be backpropagated through the stochastic sampling process, making the network trainable via standard backpropagation techniques used in deep learning.

The integrated VAE module within the MOALS framework is pivotal for reducing dimensionality and uncovering latent structures in complex multi-omics data, facilitating downstream tasks such as classification and regression with enhanced interpretability and accuracy.

The end-to-end downstream network of MOALS is capable of classification, regression, and survival prediction. Employing the method of multi-task training described in the next sections, every downstream task that fits is possible to train individuals according to one of these categories or in conjunction with other downstream tasks. To perform classification-related downstream tasks, like (Control & Target) type categorization, and site of motor onset classification, a multilayer completely linked network. The categorization downstream network's output dimensionality was set to the number of categories. An analogous network was linked to the DEM for regression, but just one neuron was maintained in the outlet layer to forestall the desired scalar amount. There is a more complex downstream network for predicting survival, which will be covered in greater detail in the next section. To make this low-dimensional latent representation even more regular, the downstream networks use the DEM to discover the omics embeddings associated with certain downstream activities and use that information in the module. Using downstream modules, from omics data, a single good-educated multi-task MOALS network can rebuild a complete diagnostic, predictive, and demographic profile.

#### 2.3.2 Learning strategy

The joint loss function, like the overall structure, has two key elements: the losses of deep embedding plus tasks in the downstream sector. For every omics profile type, $x_{j}$ is used to signify the input profile, and $x_{j}^{\prime }$ is used to denote the reconstructed profile matching that input profile, where M is different sorts of omics and the index is j. In order to calculate the deep embedding loss, we use the formula provided in Equation (5):

(5) $$L_{embed}=\frac{1}{M}\sum_{j=1}^{M}BCE\left(\;x_{j},x_{j}^{\prime }\right)+D_{KL}(N\left(\mu ,\sigma \right)||N\left(0,I\right))$$

For comparison, BCE is the binary cross-entropy, while KL divergence measures the difference between a learnt distribution and a standard Gaussian one. A **classification task**'s loss function is shown in Equation (6):

(6) $$L_{classification}=CE\left(y,y^{\prime }\right)$$

Suppose that the predicted label $y^{\prime }$ is equal to the cross-entropy loss (**CE**), and that the true label *y* represents the anticipated label. The **regression task**'s loss function is the same as the classification task's loss function, as shown in Equation (7):

(7) $$L_{regression}=MSE\left(y,y^{\prime }\right)$$

In this case, MSE stands for the average squared difference between the actual and predicted values.

MOALS was used to construct three training stages that made use of the aforementioned loss mechanisms. This was a period in which the deep embedding module was the exclusive focus, hence it was unsupervised in the beginning. This training phase solely used backpropagation to improve the deep embedding loss and only made minor adjustments to those parameters based on the gradients. While the downstream networks were being trained, the previously trained embedding network was fixed. Only the downstream networks were updated during this phase, and the total downstream loss was backpropagated.

#### 2.3.3 Survival function strategy

The **survival function**, is defined as shown in Equation (8):

(8) S(t)=P[T>t]

where **T** denotes the time elapsed during sample acquisition and the time of event happening. The survival function demonstrates the probability that the death (as the failure event) has not happened by time t. The mentioned function can be measured via Equation (9):

(9) $$h\left(t\right)=\lim_{dt\to 0}\frac{P[t\leq T<t+dt|T\geq t]}{dt}$$

This shows the instantaneous frequency with which the unsuccessful event occurs. High hazard numbers denote a high risk of death at the time t indicated by the number. It is rare to use the original hazard function in its original form; instead, the risk score for each sample x is calculated by the following formula, as shown in Equation (10):

(10) $$r\left(x\right)=\sum_{i=1}^{m}h\left(t_{i},x\right)$$

It is not only necessary to use the omics data *x*, a survival predicting downstream network, as well as the event time T and the event indication E. When a failure happened during the study, the indicator was set to 1, and when it didn't, it was set to 0, a procedure known as censoring. Time T is the interval between sample collection and the subject's last contact in the case of censorship.

#### 2.3.4 Multi-task learning

For application in the downstream task of survival prediction, the MOALS architecture was modified from the multi-task logistic regression (MTLR) model. Firstly, the time axis was split into m time intervals ${\left\{l_{i}\right\}}_{i=1}^{m}$. Time was taken into account as $l_{i}=[t_{i-1},t_{i})$, with $t_{0}=0$ being zero and $t_{m}\geq max\left(T\right)$ being the maximum allowed value. The hyperparameter *m* denotes the number of time periods that are included in the calculation. Increased precision comes at the expense of processing resources. A multi-layer fully connected network underpins our survival prediction system, and the output layer has the dimension of the number of time intervals. Consequently, we get an m-dimensional vector $y^{\prime }=\left[y_{1}^{\prime },y_{2}^{\prime },...,y_{m}^{\prime }\right]$ from our survival prediction network. At time point $t_{i}$, the survival label for each subject was kept as an m-dimensional vector $y=\left[y_{1},y_{2},...,y_{m}\right]$, with name $y_{i}$ denoting the subject's survival status. Sample *x* has the following conditions, and the probability of finding y with the network parameters *θ* is formulated by Equation (11):

(11) $$P_{\theta }\left(y|x\right)=\frac{exp\left(\sum_{i=1}^{m}y_{i}y_{i}^{\prime }\right)}{\sum_{j=0}^{m}exp\left(\sum_{i=j+1}^{m}y_{i}^{\prime }\right)}$$

The goal of this survival network is to find a set of variables *θ* that maximizes log-likelihood; consequently, the loss function for the survival prediction function is written as shown in Equation (12):

(12) $$L_{survival}=-\sum_{i=1}^{m}y_{i}y_{i}^{\prime }+log\sum_{j=0}^{m}exp\left(\sum_{i=j+1}^{m}y_{i}^{\prime }\right)$$

It can be implemented straight to the survival component and is incorporated into MOALS's joint loss function. As an alternative to training each downstream network in MOALS separately, several downstream networks in MOALS simultaneously trained using the joint loss function of the downstream tasks. This resulted in an integrated model capable of reconstructing a comprehensive phenotypic profile for each individual, as shown in Equation (13):

(13) $$L_{down}=\frac{1}{k}\sum_{k=1}^{K}W_{k}L_{down_{k}}$$

The loss associated with each function is denoted by the letter $L_{{\mathrm{down}}_{k}}$, and the weight is indicated by the $W_{k}$ that might be explicitly set as hyperparameters or utilized as trainable parameters during the training procedure. The last step required calculating and backpropagating the total loss function defined in Equation after pre-training the embedded and downstream networks independently (13). During this last training step, the whole MOALS network, including the DEM and downstream task, was fine-tuned to maximize performance.

The multi-task optimization approach gradient normalization (GradNorm) is adjusted to presented MOALS architecture to balance the optimization of varied workloads. The weights for each downstream loss are different for each training iteration. When a task's gradients are either too large or too little, GradNorm penalizes the network, ensuring that all tasks learn at a consistent rate. For starters, the gradient norm of each subsequent job is derived using the Equation (14):

(14) $$G_{\theta }^{\left(k\right)}={\left\|{▽}_{\Theta }W_{k}L_{down_{k}}\right\|}_{2}$$

In witch *θ* is the parameters of the DEM of MOALS's last encoding layer are. The mean gradient norm for all tasks can therefore be determined as shown in Equation (15):

(15) $$\overset{¯}{G_{\theta }}=\frac{1}{k}\sum_{k=1}^{K}G_{\theta }^{\left(k\right)}$$

where **K** represents the number of subsequent tasks. The following definition applies to each task's relative inverse training rate, as shown in Equation (16):

(16) $$r_{k}=\frac{{\overset{˜}{L}}_{{\mathrm{down}}_{k}}}{\frac{1}{k}\sum_{k=1}^{K}{\overset{˜}{L}}_{{\mathrm{down}}_{k}}}$$

where ${\overset{˜}{L}}_{down_{k}}=L_{down_{k}}/L_{dow{n_{k}}_{0}}$ which it is the difference between the current loss and the loss the downstream task k experienced initially. In that case, the GradNorm loss is defined as follows in Equation (17):

(17) $$L_{grad}=\sum_{k=1}^{K}{\left|G_{\theta }^{\left(k\right)}-\overset{¯}{G_{\theta }}\times r_{k}^{\alpha }\right|}_{1}$$

where *α* is the hyperparameter corresponding to the strength required to reduce tasks to a common training rate. During each training iteration, a separate backpropagation process was run on $L_{\mathrm{grad}}$, which was utilized only to update $W_{k}$.

### 2.4 Models' training and evaluating procedure

In this section, a brief description of the MOALS is presented as our proposed algorithm. MOALS implements PyTorch's deep learning library for a multi-omics ALS prediction. During the training and testing of the presented platform, the separation was conducted in a stratified manner to keep the proportion of each class; five-fold cross-validation of the train-validate data optimized the developed architecture and other hyperparameters for MOALS. Moreover, accuracy, precision, recall, and f1-score were selected as three different metrics to evaluate the performance of the proposed algorithm. It should be mentioned that the network architecture is fully connected through all layers. Besides, in this paper, shallow machine learning models also have been considered. The findings prove that there is essential to implement a deep network to obtain a significant recall. Also, the GradNorm algorithm is applied to optimize the network parameters with an initial learning rate of 0.02 and a decay of 2e-4. In a batch size of 32 and in over 200 epochs, the optimization process was conducted. The hyperparameters used to train this model were listed in Table 2.

**Table 2:** Hyper-parameters used in the model.

Hyper-parameter	Value
Latent dimension	128
Learning rate	1e-3
Batch size	32
Epoch number—unsupervised	50
Epoch number—supervised	100

With respect to multi-omics approaches and for each omics individually, the platform's network architecture is optimized. It should be noted that as well as for multi-omics one, variables are optimized separately for each omic. Next, MOLAS is applied to the test data to determine the performance of our approach. However, it should be highlighted that, for these samples, the authors utilize the candidate genes and the whole gene expression data individually. Moreover, a GPU with a 12-gigabyte capacity was utilized in this paper to develop and test the proposed algorithm.

### 2.5 A comprehensive comparison between different machine learning methods

By considering test data during the assessment of MOALS, its functionality has been evaluated with other algorithms. Three different algorithms (SVM, random forest, and fully connected neural network) were taken into account for mentioned comparison. To conduct a dimension reduction, UMAP and PCA have been utilized for each mentioned algorithm. Then, a cross-validation method is implemented to optimize hyperparameters, and finally, the performance of examined dataset is presented. To explore a non-linear boundary and consequently maximize the margin between two different clusters, SVM, as a well-known binary classification method is implemented. Also, a radial basis function is implemented as SVM kernel and its coefficient is equal to 0.001.

Moreover, it is useful to highlight some points related to features of the random forest algorithm. This algorithm is so popular, too. It is an applied machine learning method that generates multiple decision trees and blends their individual categories to reach a final classification. A higher accuracy has a tight relationship with the number of decision trees. However, increasing the number of trees has a negative impact on the training time and decreases the train speed. In the current study, 100 trees are implemented with a maximum depth of 5 and at most 100 features.

The neural network that is one of the implemented algorithms that has been selected to carry out a comparison between its performance with our proposed algorithm includes a series of fully connected layers that connect every neuron in one layer to another one in the other layer. The structure agnostic is the most significant feature of this method. In fact, in this method, no special perception is needed in the input data. In the current study, three hidden layers with ‘relu’ activation function apart from input and output layers are implemented.

With considering three different assumptions about the data in the algorithm, a dimension reduction method (UMAP) that has been founded based on them is implemented in this investigation. It should be mentioned that the data is uniformly distributed on the Riemannian manifold; The mentioned metric is approximately fixed and locally connected with respect to its position. After considering all mentioned points, it is possible to apply a fuzzy topological structure in order to simulate the manifold. Searching for a low-dimensional projection of the data that has the nearest direction equivalent to the fuzzy topological structure. It should be emphasized that 10 and 0.05 are defined for the values of the number of neighbors and the minimum distance, respectively.

PCA can significantly capture the variation present in the data with fewer parameters and provides information on the whole structure of the evaluated dataset. This action is conducted by the mentioned algorithm using linear combinations of parameters to synthase orthogonal axes. In the current manuscript, four components are considered as variables for generating the orthogonal axes.

## 3 Results and discussion

### 3.1 Identification of ALS pathway correlated genes

We performed unsupervised clustering on the RNA expression profiles to discover groups of genes with similar expression patterns in ALS samples relative to healthy controls. The main objective of this clustering was not solely to pinpoint genes directly associated with ALS but to explore a broader spectrum of biological pathways potentially contributing to ALS pathogenesis. This comprehensive approach aids in understanding the complex network of interactions and the potential overlapping pathways that could influence ALS and other neurodegenerative diseases.

For each cluster of genes identified by the algorithm, we conducted a pathway enrichment analysis. This analysis crucially allows us to identify not just the direct pathways like ALS but also other significant pathways that might be mechanistically linked. These include pathways related to neurodegeneration, cellular stress responses, and protein homeostasis, which are vital for understanding the broader biological context of ALS.

We tested different numbers of clusters (6-12) for clustering of gene expression, finding that irrespective of the number of clusters, at least one cluster was consistently associated with ALS. This not only demonstrates the robustness of our clustering approach but also supports the hypothesis that molecular signatures of ALS are strongly represented in the dataset. To visually represent this, we extracted genes that appeared in any detected ALS KEGG pathways enriched clusters and identified 9847 genes for downstream analysis.

It is important to note that our clustering approach also highlighted other pathways with even more significant p-values, such as Endocytosis. This finding underscores the multi-faceted nature of neurodegenerative diseases where multiple biological processes are often interconnected. The identification of pathways like Endocytosis with better p-values than ALS itself suggests potential upstream or parallel processes that could influence or be influenced by ALS pathology.

The Endocytosis pathway plays a crucial role in cellular homeostasis by mediating the internalization and recycling of cell surface receptors, lipids, and other molecules. Dysregulation in endocytosis has been implicated in the pathogenesis of neurodegenerative diseases, including ALS [28]. In ALS, defective endocytosis could lead to impaired clearance of misfolded proteins and other cellular debris, contributing to neuronal damage. This pathway's significance in ALS is underscored by its strong association with other neurodegenerative processes, suggesting that alterations in endocytic trafficking may be a common mechanism in ALS and other neurodegenerative disorders.

The endoplasmic reticulum (ER) is critical for proper protein folding and processing. In ALS, mutations in proteins involved in ER stress response, such as VAPB, have been shown to disrupt ER homeostasis, leading to the accumulation of misfolded proteins and triggering the unfolded protein response (UPR) [29]. Persistent UPR activation can lead to neuronal apoptosis, contributing to the progressive loss of motor neurons observed in ALS patients. The identification of this pathway emphasizes the importance of protein homeostasis in ALS and highlights potential therapeutic targets aimed at modulating ER stress responses.

Ubiquitin-mediated proteolysis is essential for the degradation of damaged or misfolded proteins via the ubiquitin-proteasome system (UPS). In ALS, disruptions in UPS have been reported, leading to the accumulation of ubiquitinated protein aggregates in motor neurons, a hallmark of the disease. This pathway's involvement in ALS is supported by the frequent observation of ubiquitin-positive inclusions in post-mortem ALS tissues. Understanding how ubiquitin-mediated proteolysis is compromised in ALS could provide insights into disease mechanisms and inform strategies to enhance protein clearance in affected neurons [30].

Autophagy is a cellular process involved in the degradation and recycling of damaged organelles and proteins. In ALS, autophagy dysfunction is believed to contribute to the accumulation of toxic proteins and organelles in motor neurons. Enhancing autophagy has been proposed as a potential therapeutic strategy for ALS, aimed at reducing the burden of protein aggregates and promoting neuronal survival. The identification of the autophagy pathway in our analysis reinforces its critical role in ALS and provides a rationale for exploring autophagy modulators as potential treatments [31].

The overlap between ALS and other neurodegenerative diseases, such as Huntington's, Parkinson's, and Alzheimer's, suggests shared pathogenic mechanisms. Common features include the accumulation of misfolded proteins, mitochondrial dysfunction, and oxidative stress. The identification of these pathways in ALS patients supports the hypothesis that ALS may share molecular pathways with other neurodegenerative conditions. This cross-disease perspective could lead to the development of broad-spectrum therapeutics targeting these shared mechanisms [32].

Moreover, the implications of these pathways extend beyond ALS, potentially illuminating common mechanisms underlying other neurodegenerative diseases such as Alzheimer's, Parkinson's, and Huntington's diseases. The disruption of cellular trafficking in the Endocytosis pathway, the imbalance in protein processing, and the failure of protein clearance mechanisms observed in ubiquitin mediated proteolysis are not only pivotal to ALS but are also critical components in the pathology of these other diseases [33]. This suggests a shared pathological framework, where targeting these pathways could lead to broad-spectrum therapeutic strategies for multiple neurodegenerative disorders. By identifying and understanding these interconnected pathways, our study contributes to a holistic view of neurodegeneration, offering insights that could inform cross-disease therapeutic approaches.

Table 3 delineates the Enrichr-derived analyses results, illustrating a substantial association between the submitted gene list and the ALS pathway, underscored by a highly significant p-value of $1.27\times 10^{-21}$ and an adjusted p-value of $2.02\times 10^{-19}$. This statistical significance minimizes the likelihood of the association emerging by random chance, reinforcing the credibility of the biological linkage inferred. Additionally, the Odds Ratio of 2.94 elucidates that the genes within our investigated list are approximately three times more probable to align with the ALS pathway than expected by chance, with the combined score of 141.56 further attesting to the robustness and overall significance of this association.

**Table 3:** The results of ALS signaling pathways obtained by MOALS.

Name	P-value	Adjusted p-value	Odds Ratio	Combined score
Endocytosis	2.35E-24	7.48E-22	4.28	232.93
Amyotrophic lateral sclerosis	1.27E-21	2.02E-19	2.94	141.56
Protein processing in endoplasmic reticulum	9.79E-21	1.04E-18	5.35	246.54
Ubiquitin mediated proteolysis	1.38E-19	1.10E-17	6.38	277.09
Huntington disease	4.26E-17	2.72E-15	2.79	105.35
Pathways of neurodegeneration	6.55E-17	3.48E-15	2.23	83.14
Parkinson disease	1.63E-15	7.41E-14	2.96	100.94
Autophagy	3.14E-15	1.17E-13	4.71	157.32
Spinocerebellar ataxia	3.30E-15	1.17E-13	4.5	150.16
Prion disease	8.63E-12	2.48E-10	2.38	60.55
Thermogenesis	9.34E-12	2.48E-10	2.57	65.37
Alzheimer disease	8.40E-11	2.06E-09	2.01	46.59

Conversely, Table 4 encapsulates the insights gained from STRING-db, revealing that 96 out of the 352 genes analyzed are implicated in ALS, with a significant association strength of 1.73. This table corroborates the insights from Enrichr by demonstrating a significant association, further emphasized by an exceptionally minimal false discovery rate (FDR) of $5.52\times 10^{-154}$, ensuring an exceptionally high level of confidence in the accuracy and relevance of this association. The extraordinary significance of the FDR consolidates the validity of the biological connection inferred between the analyzed gene network and ALS.

**Table 4:** The results of ALS signaling pathways for first hundred candidates' genes obtained by MOALS.

Pathway	Description	Count in Network	Strength	False discovery rate
hsa03050	Proteasome	20 of 43	1.96	9.63e-30
hsa05014	Amyotrophic lateral sclerosis	96 of 352	1.73	5.52e-154
hsa04136	Autophagy - other	7 of 29	1.67	1.18e-08
hsa05017	Spinocerebellar ataxia	31 of 135	1.65	6.29E-39
hsa05012	Parkinson disease	45 of 240	1.56	1.02E-54
hsa05020	Prion disease	48 of 265	1.55	4.51E-58
hsa05016	Huntington disease	52 of 298	1.53	1.05E-62
hsa05010	Alzheimer disease	56 of 355	1.49	6.77E-66

In consolidating the insights from Table 3, Table 4, it is apparent that both Enrichr and STRING-db analyses converge on a similar conclusion, highlighting a significant association between the examined gene entities and the ALS pathway. Enrichr provides a comprehensive perspective on the statistical and biological significance of the gene list in the context of ALS, while STRING-db accentuates the interactive networks among the genes, reinforcing the biological relevance of the findings. The convergence of these analyses not only strengthens the inferred association between the analyzed entities and ALS but also propels our understanding forward, opening avenues for exploring the intricate molecular mechanisms underlying ALS.

**Figure 2:**
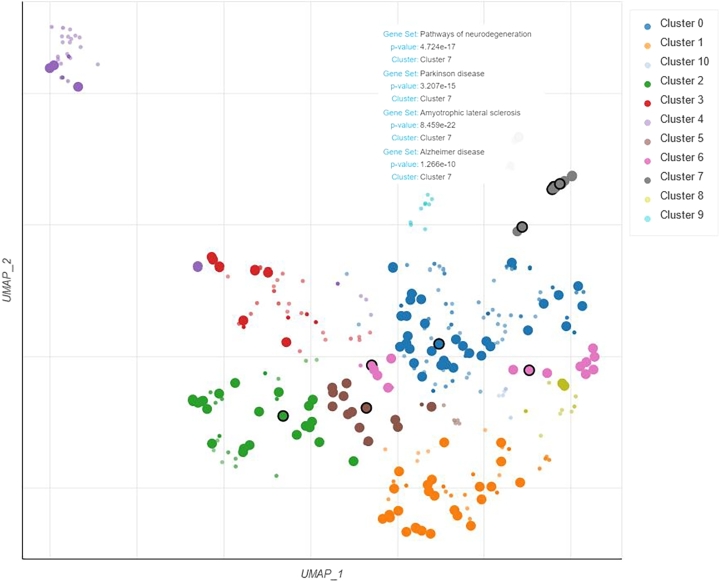
The scatter plot to demonstrate disease classification based on KEGG conducted by MOALS.

Fig. 2 displays a UMAP-based scatter plot visualizing terms from the KEGG 2021 Human gene set library extracted from the fuzzy k-means clustering algorithm. Terms, represented by points, are plotted on the first two UMAP dimensions, clustered by the Leiden algorithm based on computed TF-IDF values, allowing similar gene sets to be grouped together. Larger, black-outlined points signify terms significantly enriched, particularly relating to ALS, Parkinson's, Alzheimer's, and neurodegeneration pathways.

Next, genes were ranked according to their degree of interconnection (STRINGdb), and their association with ALS. STRINGdb detects statistically significant associations between a list of input genes and known biological pathways, and for this analysis, KEGG pathways with adjusted p-values less than 0.05 were selected.

**Figure 3:**
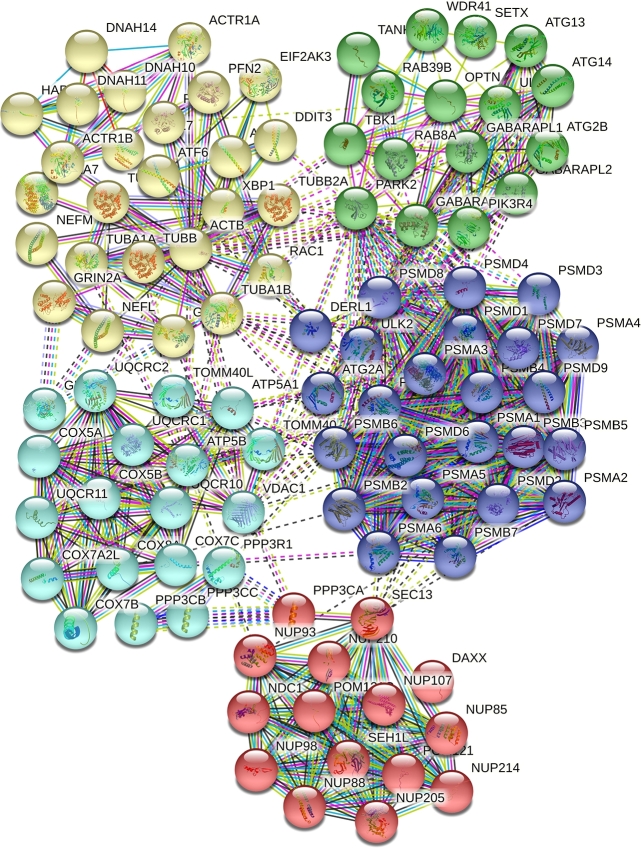
The clustering illustration of the first hundred candidates' genes by MOALS. , , , , , , , , , .

Based on current literature relating to the molecular pathways of ALS, these pathways demonstrate a remarkable degree of association. The input genes selected during the first phase of our analysis have more interconnection among each other than what normally would be expected for a random set of genes of the same size. This enrichment depicts that the mentioned parameters are at least tendentiously biologically interconnected as a group. Also, to show all the interconnection between ranked genes and argue on it, Fig. 3 has been illustrated. As can be seen in the mentioned figure, five different clusters have been detected related to ALS and other neurodegenerative diseases. The figure shows the first 100 ranked genes (as nodes) that have been connected by 604 edges. Between every two nodes, one or several edges have been recognized which are classified by some colors. Besides, co-expression that has been colored in black identifies which genes have a tendency to show a coordinated expression pattern across a group of genes.

### 3.2 Explaining multi-omics integration results

**Figure 4:**
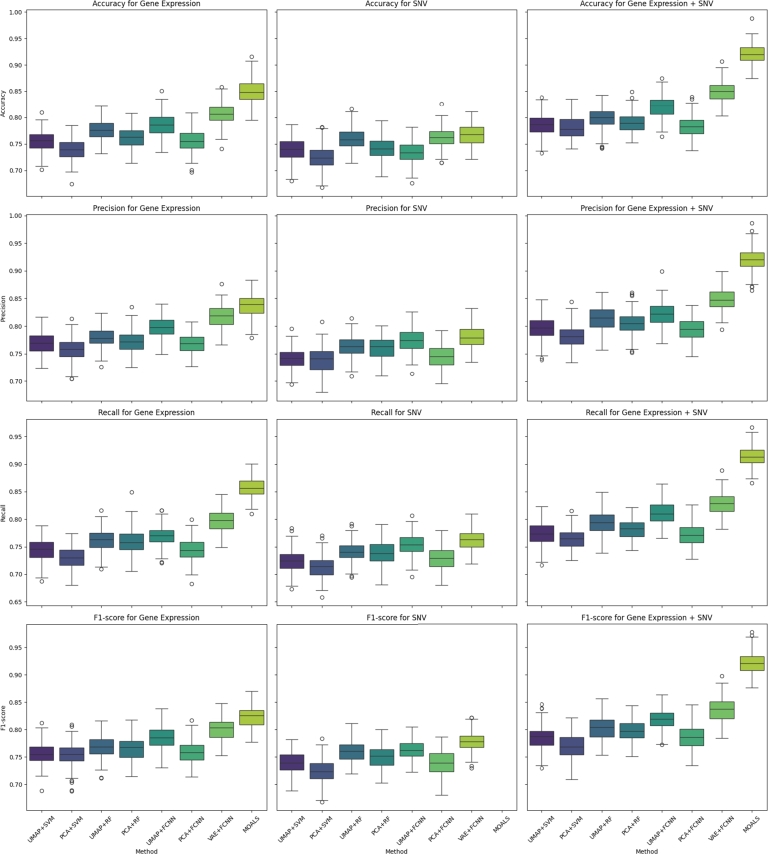
5-Fold cross-validation results for multiple classification methods on omics data.

An in-depth analysis was conducted to distinguish between ALS and healthy cases using single and multi-omic data, focusing explicitly on either RNA expression data or SNV data. To evaluate the stability and performance consistency of the classification models, a comprehensive five-fold cross-validation analysis was performed. The results, illustrated in Fig. 4, include boxplots for accuracy, precision, recall, and F1-score across the validation folds, stratified by the classification methods and omic data types—Gene Expression, SNV, and their combination. Additionally, 95% confidence intervals were calculated for each performance metric, providing a more accurate representation of variability across folds. The effect sizes (Cohen's d) were also computed to assess the practical significance of the observed differences between ALS and control groups, with values ranging between 0.87 and 1.16. This range indicates a medium to large effect size, underscoring the practical relevance and robustness of the classification models in effectively distinguishing between ALS and healthy cases.

Key observations from the boxplots include notable fluctuations in model performance metrics which may indicate variability in model robustness or adaptive responses to the integration of multi-omic data. These detailed distributions provide deeper insights into the predictive stability of each method beyond the mean performance metrics typically reported. Notably, the MOALS method consistently demonstrated superior performance across all metrics, particularly in the Gene Expression + SNV category, where it achieved the highest median scores and showed relatively tight interquartile ranges, indicating less variability and higher reliability in comparison to other methods.

Relating these insights to the consolidated results presented in Table 5, it is evident that the tabulated data encapsulates the average performance metrics from the cross-validation exercise. While the table effectively compares the methodological efficacies, the boxplots enrich this comparison by detailing the range and consistency of performance across multiple experimental runs, thus offering a holistic view of each method's efficacy and reliability. The standout performance of MOALS, as highlighted in the cross-validation plots, underscores its robustness and underscores its potential as a highly effective tool for integrating multi-omic data in the classification of ALS.

**Table 5:** Classification results based on six classification methods applied by single omic and multi-omics approaches.

	Gene Expression				SNV				Gene Expression + SNV			
	Accuracy	Precision	Recall	F1-score	Accuracy	Precision	Recall	F1-score	Accuracy	Precision	Recall	F1-score
UMAP+SVM	0.7564	0.7663	0.7438	0.7573	0.7367	0.7415	0.7273	0.7368	0.7846	0.7938	0.7743	0.7847
PCA+SVM	0.7406	0.7562	0.7305	0.7497	0.7252	0.7369	0.7161	0.7234	0.7796	0.7816	0.7653	0.7713
UMAP+RF	0.7761	0.7812	0.761	0.7704	0.7589	0.7612	0.7414	0.7598	0.801	0.8116	0.792	0.8002
PCA+RF	0.7656	0.7729	0.759	0.7645	0.7414	0.7599	0.7371	0.7486	0.79	0.8019	0.7809	0.7971
UMAP+FCNN	0.7824	0.7979	0.7702	0.7864	0.7628	0.7749	0.7561	0.7618	0.8183	0.823	0.8076	0.8176
PCA+FCNN	0.7564	0.7663	0.7438	0.7573	0.7367	0.7415	0.7273	0.7368	0.7846	0.7938	0.7743	0.7847
VAE+ FCNN	0.8048	0.8176	0.7965	0.8035	0.7706	0.7826	0.7638	0.7762	0.8505	0.8476	0.8255	0.8371
MOALS	0.8485	0.8374	0.8566	0.8214					**0.9206**	**0.9186**	**0.9148**	**0.9211**

The comparative analysis delineates the performance of various dimensionality reduction techniques when coupled with machine learning classifiers, specifically in the context of Gene Expression data, SNV data, and their integration. Performance is quantified via accuracy, precision, recall, and F1-score, offering a comprehensive evaluation of each method's efficacy.

Upon examining the performance of UMAP with SVM (UMAP+SVM), one observes a moderate level of accuracy at 0.7564 for Gene Expression data, precision at 0.7663, recall at 0.7438, and an F1-score of 0.7573. This suggests a balanced classification capability. However, the application of this combination to the integrated dataset yields improved results, with the accuracy and F1-score elevating to 0.7846 and 0.7847, respectively. However, an intriguing enhancement in performance is observed when SVM is applied to the integrated Gene Expression and SNV dataset, suggesting that SVM classifiers benefit from a richer feature space that encapsulates a more diverse biological signal.

The PCA+SVM combination, while similar in approach to UMAP+SVM, records a slightly reduced accuracy of 0.7406 and an F1-score of 0.7497 for Gene Expression data. The metrics for the combined dataset are marginally lower than those for UMAP+SVM, with an accuracy of 0.7796 and an F1-score of 0.7713, reaffirming UMAP's superior feature extraction capability for SVM classifiers.

Moving to ensemble methods, UMAP paired with Random Forest (UMAP+RF) shows an appreciable accuracy of 0.7761 and an F1-score of 0.7704 for Gene Expression data, which is a marked improvement over SVM-based methods. The amalgamation of Gene Expression and SNV data under UMAP+RF further improves accuracy to 0.8010 and the F1-score to 0.8002, suggesting an efficient harnessing of combined data features.

For neural network-based classifiers, UMAP+FCNN displays notable efficacy, particularly in the integrated dataset, where the accuracy reaches 0.8183 and the F1-score climbs to 0.8176. This combination outperforms all other non-neural network classifiers, indicating FCNN's superior ability in modeling complex data interactions. A substantial leap in performance is evident with VAE+FCNN, especially for the integrated dataset, where the accuracy surges to 0.8505 and the F1-score to 0.8371. This indicates the VAE's powerful feature extraction capability in conjunction with FCNN's classification strength.

The standout performer, MOALS, which incorporates a clustering algorithm for feature selection followed by VAE+FCNN for classification, achieves the highest accuracy of 0.9206 and an F1-score of 0.9211 in the integrated dataset. These metrics are considerably higher than those of other methods, underscoring the profound impact of feature selection through clustering in enhancing classifier performance. The Multi-Omics Analysis with Latent Structures (MOALS) approach, integrating the clustering algorithm with VAE+FCNN, showcases the pinnacle of classification performance. This technique's robustness is evidenced by the top-tier metrics across all evaluated categories in the combined dataset. MOALS effectively narrows down the feature space to the most discriminative gene sets, which evidently facilitates a more refined and targeted classification process.

**Figure 5:**
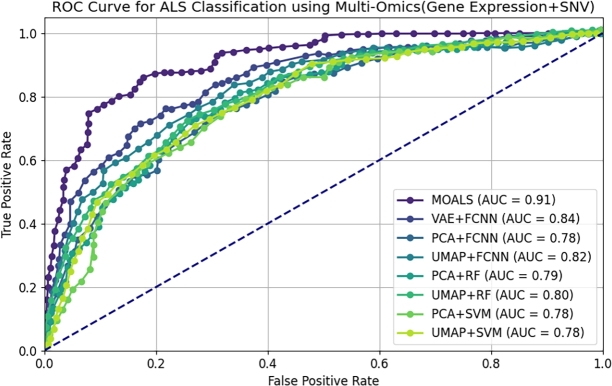
AUC-ROC curve for ALS classification using multi-omics (Gene Expression + SNV).

Fig. 5 illustrates the Receiver Operating Characteristic (ROC) curves for various classification methods, with a focus on the integrated Gene Expression and SNV dataset. The Area Under the Curve (AUC) is a critical metric for evaluating the performance of classifiers, as it provides a single scalar value to compare models. Among the methods evaluated, MOALS achieved the highest AUC of 0.91, indicating superior discriminatory power in distinguishing between ALS and control cases. This performance further solidifies the MOALS approach as the most effective model in our study, outperforming other deep learning and traditional machine learning models. The VAE+FCNN model, while performing well, recorded a lower AUC of 0.84, followed by UMAP+FCNN at 0.82, reinforcing the advantages of multi-omics data integration combined with the MOALS methodology. The remaining models exhibited moderate performance, with AUC values ranging from 0.78 to 0.80, highlighting the impact of model selection and data integration strategies on predictive accuracy.

**Figure 6:**
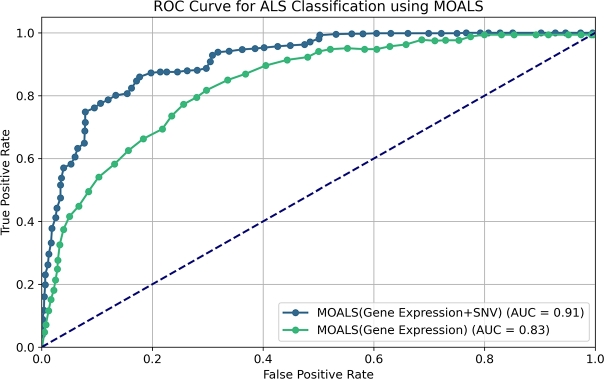
ROC curve comparison for ALS classification using MOALS with single-omic (Gene Expression) and multi-omic (Gene Expression + SNV) data.

Fig. 6 presents a comparison of ROC curves between the MOALS model using single-omic data (Gene Expression) and the MOALS model using integrated multi-omic data (Gene Expression + SNV). The results clearly indicate that the multi-omic approach significantly outperforms the single-omic approach, as evidenced by the higher AUC value (0.91 for multi-omic vs. 0.83 for single-omic). This highlights the substantial improvement in classification accuracy that can be achieved by incorporating diverse data types, thus providing a more comprehensive understanding of the underlying biology in ALS.

In conclusion, the study underscores the crucial role of integrating multi-omic data with dimensionality reduction and machine learning techniques in analyzing complex biological data. The findings highlight how deep learning, when combined with advanced dimensionality reduction strategies and multi-omic integration, can effectively reveal the subtle biological dynamics underlying complex diseases like ALS, paving the way for breakthroughs in precision medicine.

An in-depth analysis was conducted to distinguish between ALS and healthy cases using single and multi-omic data, focusing explicitly on either RNA expression data or SNV data. To elucidate the superiority of MOALS, a comprehensive comparison with existing models such as PCA+FCNN, UMAP+FCNN, and other commonly utilized models in ALS research is provided. This comparison extends beyond performance metrics to include methodological differences, highlighting how MOALS's integrative approach to multi-omics data provides a more robust and accurate prediction model. Specifically, the integration of clustering algorithms and VAE+FCNN allows MOALS to effectively handle the heterogeneity and complexity of multi-omic data, leading to improved prediction accuracy and reliability.

The use of advanced dimensionality reduction techniques combined with deep learning architectures differentiates MOALS from traditional models that may not fully exploit the potential of integrated multi-omic datasets. Furthermore, the implementation of cross-validation in MOALS is detailed, explaining how this was adapted to manage the complexities of multi-omic data, including the stratification of data across different omic types to ensure that the validation process is robust and reflects the true predictive power of the model.

These methodological advancements are crucial for understanding why MOALS performs better than other models, as it not only leverages genetic information but also incorporates epigenetic, transcriptomic, and proteomic data, providing a holistic view of the disease pathology. In summary, the innovative approach of MOALS sets a new standard in the field by not just incrementally improving over existing models but by redefining what is possible in the prediction and understanding of complex diseases like ALS.

Our findings suggest that the MOALS model, with its superior diagnostic accuracy and robustness in integrating multi-omic data, could be effectively incorporated into existing clinical diagnostic pathways for ALS. Specifically, we propose that the model be used as a complementary tool alongside traditional clinical tests, such as neuroimaging and electrophysiological studies, to enhance diagnostic precision. By incorporating MOALS into the diagnostic workflow, clinicians may achieve faster and more accurate identification of ALS, enabling earlier intervention and potentially improving patient outcomes. This integration could also lead to more efficient utilization of healthcare resources by reducing the need for multiple confirmatory tests, thereby streamlining the diagnostic process. Furthermore, the predictive power of MOALS extends beyond diagnosis; it also demonstrates a significant capability in related predictive tasks, such as estimating the onset age of ALS symptoms and survival prediction.

**Figure 7:**
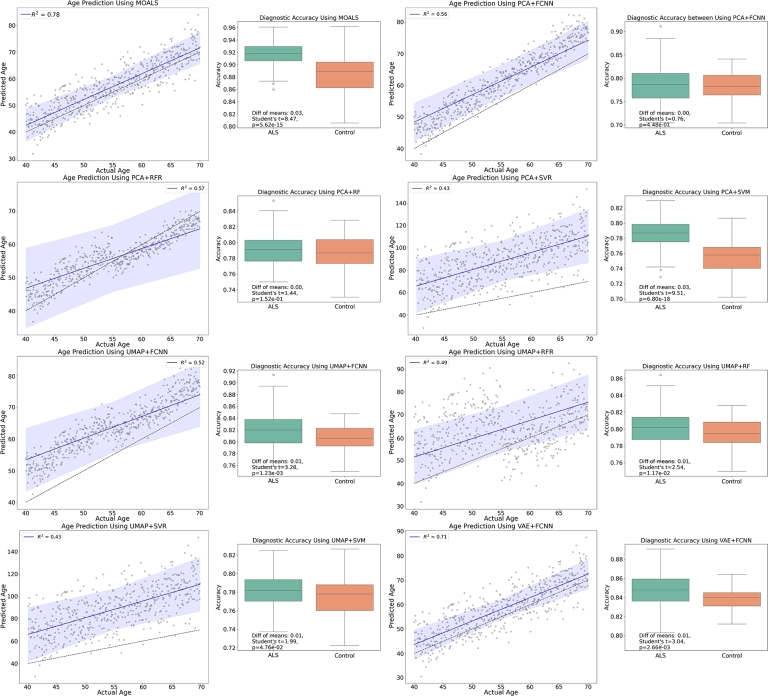
Comparison of different models for age prediction and diagnostic accuracy.

Fig. 7 provides a comprehensive display of regression lines and diagnostic accuracy for various models. In the regression plots, the MOALS model is distinguished by achieving the highest $R^{2}$ value of 0.78, indicating a strong linear correlation and capturing about 78% of the variance in age data. This high $R^{2}$ value highlights MOALS' superior capability in integrating complex multi-omic data, which significantly surpasses other models like VAE+FCNN with an $R^{2}$ of 0.71, and models like UMAP+RFR and PCA+RFR, which show moderate fits with $R^{2}$ values of 0.49 and 0.57, respectively. The confidence intervals around the regression lines in MOALS' plot are notably narrower, pointing to its higher precision in age predictions. This contrast is evident when compared to PCA+FCNN and UMAP+FCNN, where broader intervals suggest greater prediction variability.

In addition to regression analysis, the box plots in Fig. 7 highlight the diagnostic accuracy between ALS and Control groups. Here, statistical analysis, particularly t-tests, shows significant differences in the means of ALS and Control groups, underscoring the diagnostic reliability of the models. Despite the unbalanced sample sizes between the ALS and control groups, the diagnostic accuracy distributions depicted in Fig. 7 demonstrate a remarkable consistency across both groups. The box plots reveal that the median accuracy levels for ALS and Control are closely aligned within the context of each model, particularly for the MOALS model. This suggests that despite the variability in group sizes, the model's performance in distinguishing between ALS and Control remains robust, ensuring reliable diagnostic outcomes.

This finding is particularly significant as it underscores the model's capability to generalize well across different group sizes without a loss in predictive accuracy. It highlights the efficacy of the model in handling imbalanced datasets, which is a common challenge in medical diagnostics. Such resilience in performance, evidenced by the narrow interquartile ranges and similar medians in the box plots, supports the utility of the model in clinical settings where the proportion of cases to controls may not always be balanced. The statistical robustness of MOALS, corroborated by the t-tests indicating significant differences between the ALS and Control groups, further enhances the credibility of the model. This statistical rigor, combined with the consistent accuracy across diverse group sizes, provides compelling evidence of the model's suitability for real-world applications, promising enhanced diagnostic precision in the clinical diagnosis of ALS.

Table 6 corroborates these findings by quantifying the relative error indices—RMSE, MAE, and MedAE—across the models. MOALS' remarkable reduction in all error metrics, especially in a multi-omics environment, is clearly delineated, reaffirming its efficacy in the predictive analytics realm. This detailed examination accentuates the critical role of leveraging diverse omic data to enhance the accuracy and reliability of biomedical predictions, firmly establishing MOALS as a groundbreaking tool in predictive methodologies.

**Table 6:** The first symptom age prediction results based on six prediction methods applied by single omic and multi-omics approaches.

	Gene Expression				SNV				Gene Expression + SNV			
	MedAE	MAE	RMSE	*R*^2^	MedAE	MAE	RMSE	*R*^2^	MedAE	MAE	RMSE	*R*^2^
UMAP+SVR	8.839	11.492	14.830	0.41	10.482	11.592	14.482	0.41	9.953	10.884	13.861	0.43
PCA+SVR	8.919	10.592	14.005	0.40	9.963	10.836	13.936	0.42	9.829	10.563	13.685	0.43
UMAP+RFR	8.899	9.971	12.991	0.45	8.928	9.949	13.002	0.48	8.936	9.925	12.893	0.49
PCA+RFR	8.850	9.283	12.629	0.54	8.994	9.385	12.693	0.52	8.317	9.623	12.317	0.57
UMAP+FCNN	8.965	10.037	13.199	0.51	8.842	9.513	12.839	0.50	8.629	9.666	12.582	0.52
PCA+FCNN	8.482	9.472	11.973	0.55	8.328	8.873	11.284	0.61	8.094	9.434	11.119	0.56
VAE+FCNN	8.194	8.548	10.913	0.64	**7.893**	**8.361**	**9.952**	**0.68**	6.625	8.242	10.619	0.71
MOALS	**7.683**	**8.192**	**9.837**	**0.69**					**6.015**	**7.158**	**9.609**	**0.78**

A meticulous examination of the table reveals that the MOALS model, a proposed multi-omics approach, notably outperforms the other models, showcasing the least error across all the enlisted metrics—MedAE, MAE, RMSE, and achieving the highest $R^{2}$ of 0.78 in the Gene Expression + SNV approach. It demonstrates superior analytical accuracy and reliability, with errors reduced by 10.14 percent, 15.14 percent, and 10.51 percent in MedAE, MAE, and RMSE respectively, when compared to the best-performing multi-omics approach developed using alternative algorithms.

Delving deeper into the specifics, the MOALS model exhibits unambiguous supremacy, attaining the lowest MedAE of 6.015, MAE of 7.158, and RMSE of 9.609, underscoring its enhanced predictive precision and reliability in estimating the first symptom age in ALS. When juxtaposed with the single omic approaches, the superior analytical finesse of the MOALS model becomes increasingly evident, especially in the context of Gene Expression and SNV data.

In contrast, while the VAE model demonstrated considerable prowess, especially in the single omic approaches, it was discernibly overshadowed by the enhanced accuracy and reduced error rates manifested by the MOALS model in the multi-omics approach, specifically in the integrated Gene Expression + SNV method. This reinforces the pivotal role of integrated multi-omic methodologies in achieving heightened precision and reliability in predictive analytics, surpassing the capabilities of singular omic data analyses.

Furthermore, the PCA+FCNN and UMAP+FCNN models, despite their commendable performance in both single and multi-omic approaches, were unable to match the elevated levels of analytical accuracy and reduced error margins achieved by the MOALS model, thereby reiterating the superior diagnostic capabilities of the latter.

In conclusion, the results derived from the comparison accentuate the paramount importance and enhanced diagnostic proficiency of the developed multi-omics model, MOALS, in predicting the onset of ALS symptoms with refined precision and minimized error, validating its significant potential as a groundbreaking tool in biomedical research. The holistic integration of diverse omic data in the MOALS model unequivocally contributes to its augmented reliability and precision, setting a new benchmark in the contemporary spectrum of predictive methodologies.

Table 7 illustrates a meticulous comparative evaluation focusing on survival prediction, employing various methods applied by both single omic and multi-omics approaches. The evaluated models are juxtaposed based on two pivotal metrics: the Concordance index (C-index) and Integrated Brier Score (IBS), both quintessential for appraising survival prediction tasks. A C-index value of 1 represents the epitome of prediction accuracy, signifying an excellent model, while a value of 0.5 symbolizes a model performing no better than random. Concurrently, the accuracy of a predicted survival function at specific time points, represented by IBS, ranges between 0 and 1, with lower scores depicting higher levels of model accuracy.

**Table 7:** The survival prediction results in different methods applied by single omic and multi-omics approaches.

	Gene Expression		SNV		Gene Expression+SNV	
	C-Index	IBS	C-Index	IBS	C-Index	IBS
UMAP+CoxPH	0.617	0.209	0.627	0.208	0.693	0.201
PCA+CoxPH	0.592	0.224	0.616	0.220	0.671	0.216
UMAP+random survival forest	0.614	0.220	0.629	0.219	0.655	0.217
PCA+random survival forest	0.659	0.211	0.673	0.199	0.685	0.204
VAE(Selected Gene)	0.697	0.200	0.719	0.195	0.745	0.188
MOALS	0.777	0.180			**0.837**	**0.121**

From a detailed viewpoint, the MOALS model conspicuously stands out, showcasing an unparalleled C-index value of 0.837, the highest across all examined models and approaches, coupled with the most optimal IBS value of 0.121, reinforcing the enhanced predictive accuracy and reliability of the proposed multi-omics model. These figures not only accentuate the formidable precision of MOALS in predicting survival but also illuminate its superior analytical reliability in comparison to the other enlisted models, especially within the realm of integrated Gene Expression + SNV approach.

Delving deeper, VAE(A) also demonstrated commendable prowess, reflecting a substantial C-index of 0.719 and an impressive IBS of 0.195 within the SNV method, marking it as a noteworthy contender in the landscape of survival prediction models. However, even with its significant precision, it doesn't overshadow the supremacy of the MOALS model in the multi-omics context, further validating the extensive capabilities of MOALS in offering a more nuanced and comprehensive analytical perspective.

Analyzing the results within the confines of single omic approaches, it is unequivocal that the models exhibit varying degrees of accuracy and reliability, with MOALS achieving a pioneering C-index of 0.777 and the most favorable IBS of 0.180 in Gene Expression. This underscores MOALS's superior predictive capabilities and higher levels of accuracy, even in singular omic data analyses, bolstering its standing as a multifaceted analytical tool.

In light of the above elucidation, the enhanced functionality and the groundbreaking precision of the proposed multi-omics model, MOALS, are conspicuously ratified. It sets a novel paradigm in the domain of survival prediction, offering a more refined, holistic, and high-resolution insight into survival prediction methodologies. The enhanced C-index and optimized IBS values exhibited by MOALS emphasize its unparalleled capability to provide more nuanced, reliable, and precise survival predictions, solidifying its potential as an innovative and pioneering instrument in advanced biomedical research and analytics.

## 4 Limitations of the study and proposed framework for future research

The insights from this study highlight the significant potential of the MOALS model in unraveling the complex molecular landscapes and gene interrelationships associated with Amyotrophic Lateral Sclerosis (ALS). Through the integration of multi-omics data and advanced machine learning techniques like Variational Autoencoders (VAEs), this research has made considerable progress in identifying key pathways that may contribute to ALS pathogenesis. However, several limitations should be addressed, and future research directions considered to enhance the model's robustness, generalizability, and clinical applicability.

A primary challenge is the dependency on large-scale, high-quality multi-omic datasets, which are not always accessible. This limitation could lead to biased findings, as genetic and environmental factors influencing ALS can vary widely across populations. Future research should prioritize the inclusion of geographically and ethnically diverse datasets to improve the model's generalizability and relevance across different clinical settings. The current study's datasets may not fully capture global genetic diversity, limiting the assessment of the MOALS model's applicability across varied populations. To enhance generalizability, validating and refining the model using more diverse datasets is crucial. The computational complexity of the MOALS model, especially with VAEs, poses another significant limitation. The high demands for computational resources and expertise may challenge broader applications, particularly in clinical settings. Future research should focus on optimizing computational efficiency and enhancing the model's interpretability, potentially by incorporating explainable AI techniques.

While the study successfully integrates gene expression and genomic variant data, the MOALS model's full potential could be realized by adding additional omics layers, such as proteomics and metabolomics. These layers would offer a more comprehensive understanding of ALS, although integrating such diverse data types presents challenges. Addressing these could lead to even more robust models and the identification of novel biomarkers. Finally, the translational impact of the MOALS model depends on rigorous validation in clinical settings. This includes independent validation, clinical trials, and assessing the model's ability to predict clinical outcomes. Ethical and data privacy considerations must also be addressed, particularly concerning the use of sensitive genomic data. Ensuring data privacy and navigating ethical concerns are essential for the equitable application of models like MOALS in clinical practice.

## 5 Conclusion

This study developed a novel multi-omics approach to understanding the genetic underpinnings of Amyotrophic Lateral Sclerosis (ALS) using machine learning techniques. By integrating gene expression profiles and rare pathogenic genomic variants, the study identified 17,546 genes associated with ALS pathways. The Multi-Omics for ALS (MOALS) model, utilizing unsupervised clustering and a Variational Autoencoder (VAE), revealed intricate genotype-phenotype correlations within the dataset.

The MOALS model significantly outperformed traditional single-omic models, improving diagnostic accuracy by 1.7% and 6.2% compared to SNV and RNA expression-based models, respectively. These findings highlight the superiority of a multi-omic approach in capturing the complex biological interactions underlying ALS, offering a more nuanced understanding of the disease's molecular architecture.

Given its performance, the MOALS model holds potential for enhancing diagnostic precision, informing prognosis, and guiding personalized therapeutic strategies. This study underscores the importance of integrating multi-omic data in Amyotrophic Lateral Sclerosis research and contributes to uncovering the molecular mechanisms driving ALS and other complex disorders.

## CRediT authorship contribution statement

**Hima Nikafshan Rad:** Writing – review & editing, Writing – original draft, Visualization, Validation, Methodology, Formal analysis, Conceptualization. **Zheng Su:** Conceptualization. **Anne Trinh:** Writing – review & editing. **M.A. Hakim Newton:** Writing – review & editing. **Jannah Shamsani:** Formal analysis. **NYGC ALS Consortium:** Data curation. **Abdul Karim:** Investigation. **Abdul Sattar:** Supervision.

## Declaration of Competing Interest

The authors declare that they have no known competing financial interests or personal relationships that could have appeared to influence the work reported in this paper.

## Data Availability

The authors do not have permission to share data. In the context of this research, RNA-sequencing data were derived from the collaborative efforts of the NYGC ALS Consortium, with detailed records available in the NCBI's GEO database, specifically under the accession numbers GSE137810, GSE124439, GSE116622, and GSE153960. Additionally, Whole Genome Sequencing (WGS) data can be made available, but they are provided strictly on a request basis. To obtain immediate access to the most recent datasets from the NYGC ALS Consortium or to procure samples from the Target ALS Postmortem Core, it is necessary for interested researchers to fill out and submit a genetic data request form, which can be sent via email to CGND_help@nygenome.org.
